# One-Dimensional Convolutional Neural Network for Object Recognition Through Electromagnetic Backscattering in the Frequency Domain

**DOI:** 10.3390/s25226809

**Published:** 2025-11-07

**Authors:** Mohammad Hossein zadeh, Marina Barbiroli, Simone Del Prete, Franco Fuschini

**Affiliations:** Department of Electrical, Electronic, and Information Engineering “G. Marconi”, University of Bologna, 40136 Bologna, Italy; marina.barbiroli@unibo.it (M.B.); simone.delprete4@unibo.it (S.D.P.); franco.fuschini@unibo.it (F.F.)

**Keywords:** Convolutional Neural Networks, deep learning, electromagnetic backscattering, measurements, object recognition

## Abstract

Over the last few decades, the item recognition problem has been mostly addressed through radar techniques or computer vision algorithms. While signal/image processing has mainly fueled the recognition process in the past, machine/deep learning methods have recently stepped in, to the extent that they nowadays represent the state-of-the-art methodology. In particular, Convolutional Neural Networks are spreading worldwide as effective tools for image-based object recognition. Nevertheless, the images used to feed vision-based algorithms may not be available in some cases, and/or may have poor quality. Furthermore, they can also pose privacy issues. For these reasons, this paper investigates a novel machine learning object recognition approach based on electromagnetic backscattering in the frequency domain. In particular, a 1D Convolutional Neural Network is employed to map the collected, backscattered signals onto two classes of objects. The experimental framework is aimed at data collection through backscattering measurements in the mmWave band with signal generators and spectrum analyzers in controlled environments to ensure data reliability. Results show that the proposed method achieves 100% accuracy in object detection and 84% accuracy in object recognition. This performance makes electromagnetic-based object recognition systems a possible solution to complement vision-based techniques, or even to replace them when they turn out impractical. The findings also reveal a trade-off between accuracy and processing speed when varying signal bandwidths and frequency steps, making this approach flexible and possibly suitable for real-time applications.

## 1. Introduction

Different from object detection, which essentially involves any technique that provides binary information about the presence of an item in a given space region, object recognition (OR) aims at object identification [1]. OR can be carried out at two different levels: (i) class level (classification), if the goal is the recognition of the type of item among a set of categories or classes (e.g., a car or a bicycle); (ii) item level, if the task is the precise recognition of the object under observation (e.g., a specific car model among different cars). The former issue is addressed in this study. Somehow, in the middle between detection and recognition lies object characterization, which is aimed at obtaining some specific properties of an item (e.g., the material it is made of) [2]. OR has many applications, such as robot navigation, medical diagnosis, security, industrial detection, and information retrieval [1,3,4]. Moreover, the potential of OR extends to supporting individuals with visual impairments, by providing real-time information about the surrounding environment, e.g., assisting in locating personal belongings and navigating around obstacles [5].

Nowadays, the most commonly used methods for OR apply pattern recognition algorithms to images or videos, i.e., they rely on a vision-based approach [6,7,8]: these techniques process visual input from cameras or similar sensors, i.e., images, to detect and recognize objects. There are various drawbacks to vision-based OR systems including the following: (i) they demand substantial computational resources, making them sometimes unsuitable for real-time applications or deployment on devices with limited processing power and memory [9]; (ii) images may be simply unavoidable in environments where cameras are forbidden, or their quality might be sometimes compromised by some environmental factors, resulting in noise or unreliable data [10]; (iii) relying on video cameras for object recognition introduces potential security risks, as these systems are often connected to the internet, making them vulnerable to hacking. This could allow unauthorized access, and the fraudulent acquisition of images of sensitive environments or industrial processes [11].

Another potential approach is based on electromagnetic waves. As well proven and explained in spectroscopy-related literature [12], objects have a unique response to incoming electromagnetic waves, depending on their material, size, and shape. When electromagnetic waves impinge on an object, a backscattered wave is radiated all around, which is univocally related to the item, as it depends on its molecular structure and specific size, shape, and orientation [13].

The first investigations on OR date back to more than fifty years ago [14,15], when image/signal processing—e.g., applied to radar signal returned by the target object—was basically relied on to accomplish the recognition task [16,17]. Not surprisingly, machine learning (ML) techniques have been replacing signal processing in recent decades, and are likely to be the current state of the art for OR.

In this study, backscattered signals from various objects over a 1 GHz band around 38 GHz are collected and stored to build a dataset for training and testing an ML model aimed at OR. The underlying assumption is that the backscattered responses of objects within the same class will exhibit a stronger correlation compared to those from different classes, as the frequency response of these items is directly related to their physical and geometrical properties. Since ML techniques are particularly effective in catching common patterns among data, the extent to which they can be entrusted for item recognition is worthy of further investigation.

Among the multitude of available ML algorithms, a 1D Convolutional Neural Network (1D CNN) was selected for this task due to its ability to effectively capture patterns in sequential data [18,19,20]. The 1D CNN is particularly well suited for analyzing the scattering responses collected in the frequency domain across the considered classes of objects because it can learn and recognize characteristic features of the signals—such as amplitude and envelope variations over the targeted frequency range—that distinguish one object class from another. This may therefore represent an effective choice for identifying subtle differences between the frequency responses of various objects, ultimately leading to satisfactory object recognition.

The main contributions of this work are briefly listed herein:

- Employment of a 1D CNN model trained on frequency-domain backscattering responses for object detection and recognition, while most of the existing literature is focused on image-based CNNs.
- Design of an experimental framework to collect datasets from multiple realizations of objects for each considered class that represents a new challenge with respect to similar, previous works.
- Investigation on the reliability of electromagnetic-based recognition as a complementary or alternative approach to vision-based systems.
- Analyses of the impact of acquisition settings on recognition accuracy, highlighting trade-offs between performance and processing time.

The remainder of the paper is organized as follows: after a brief survey of previous studies on OR in Section 2, Section 3 explains the experimental framework, including the measurement setup, equipment, objects, and the collected data. The ML model and the performance metrics are introduced in Section 4, while Section 5 presents the results and discusses the OR performance. Some conclusions are finally drawn in Section 6.

## 2. Background on Object Recognition

Although object recognition techniques have been attracting attention in the last fifty years [14,15], investigations have been essentially limited for a long time to computer vision algorithms [6,7], as also summed up in Table 1. Nevertheless, image-based approaches to OR are often computationally heavy, and can have poor performance in case the input images are compromised (e.g., because of adverse weather conditions in outdoor applications, or in the presence of smoke or dust in industrial contexts). Moreover, they can pose privacy issues. In addition to image-driven solutions, some investigations on electromagnetic fields for OR have also been carried out (Table 1). For instance, OR at THz frequencies is explored in [21,22], ultimately to detect and identify objects (mainly made of metal or liquids) hidden behind some shielding surface (e.g., luggage scanning). This task can be effectively accomplished at THz frequency as many alleged shielding materials are actually transparent in the THz band (including most dielectrics). As a matter of fact, a THz OR would therefore turn out ineffective for the recognition of items made of such transparent materials. Since the data acquired through THz scanning are in the end converted into an image (i.e., suited to computer vision algorithms), most of the effort is actually spent in [21,22] to improve the quality of the images coming from the THz scanner, which are often affected by severe noise.

**Figure 1:**
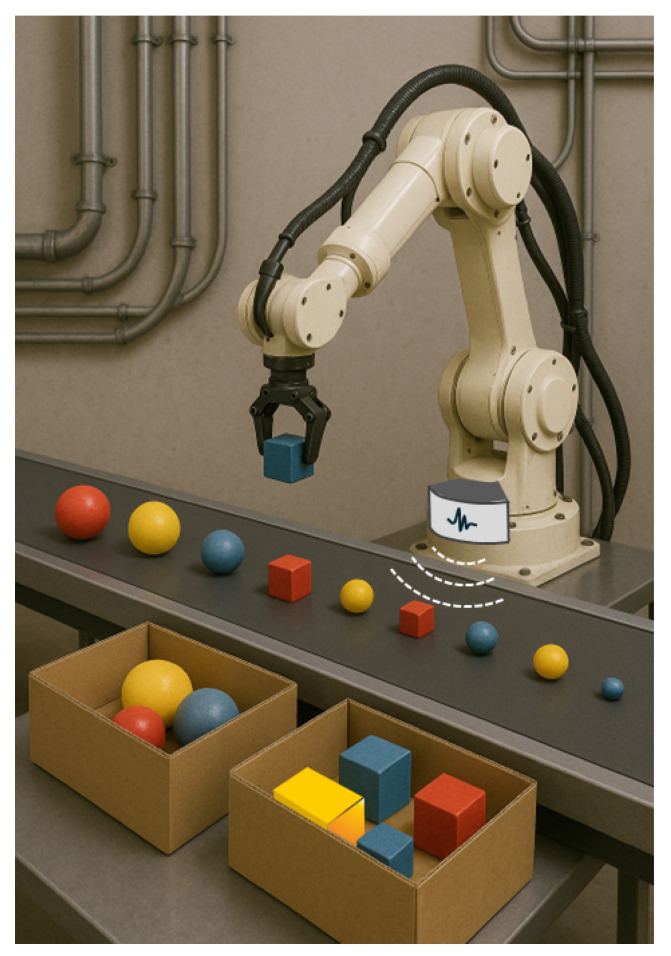
OR in an industrial context.

Although radar systems have been primarily conceived for object detection, they can also tackle the recognition task to some extent [10]. This idea is discussed in, for instance, [16,17], where the electromagnetic backscattering responses from some reference items illuminated by an ultra-wideband (UWB) radar transmitter moving all around are estimated by means of simulations. Signal processing is then applied to the collected signals for object identification. A similar procedure is applied in ground-penetrating radar (GPR) applications, where a pulse sequence is radiated by an antenna above the ground, which simultaneously gathers the backscattered response (A-scan) in the time domain from items possibly buried below the Earth surface. The antenna is usually moved along a scanning path over the ground, and the 1D, A-scan signals corresponding to the different antenna positions are then merged into a space–time backscattering profile (B-scan) that can be finally represented as a 2D image [23,24]. Post-processing and/or ML techniques are then applied to the B-scan images or—less frequently—to the 1D A-scan signal [2] to detect the presence of the item and its major characteristics (like the material it is made of), or—in the best case—to recognize it. It is worth pointing out that moving the radar antenna around the target object or along a scanning path might hardly be possible in many cases, e.g., in industrial applications, where the freedom of movement can be limited by space restriction and fast recognition might be required (see, for example, Figure 1). Other radar-based OR solutions leverage (inverse) synthetic aperture images [25], which can then be traced back to computer vision OR, or objects (micro-)Doppler signature, which means they are limited to moving targets only [25,26]. Radar automatic target recognition can also rely on high-resolution range profiles (HRRPs), which represent the spatial distribution in the range domain of the strongest sub-echoes returned by prominent scatterers on the target surface [25,27]. Recent studies have applied deep learning to HRRP-based recognition, including methods based on scattering center networks [28], multi-modal support vector data description [29], and graph neural networks for few-shot classification [30]. More recently, a noise-robust method for HRRP recognition that combines a low-rank decomposition with CNN-based classification is proposed in [28], thereby improving recognition performance under severe noise conditions. Although effective, this approach still relies on HRRP sequences obtained from large radar targets, whereas our work focuses on frequency-domain backscattered sweeps collected from smaller everyday objects. These approaches are mostly suited to large targets, where the spacing among scatterers is larger than the radar resolution (e.g., military aircraft in [31]).

In [32], a 1D CNN model for automatic target classification in complex environments is developed and exploits Doppler spectrograms to discriminate moving targets, achieving high recognition accuracy. While similar in adopting a 1D CNN architecture, the approach is tailored to Doppler-based features from dynamic scenes.

Regardless of whether the recognition task is triggered by a visual or an electromagnetic input, it is nowadays mostly addressed through machine/deep learning algorithms [21,22,26,27,31,32], which are increasingly replacing previous signal processing techniques [16,17]. In the broad framework of ML methods, CNNs are generally acknowledged as reliable solutions for OR.

**Table 1:** Survey on image-based and EM wave-based methods for object detection/recognition.

Type	Ref.	Detection/Recognition	Notes
Image-Based	[3]	Detection, Recognition	Deep learning for object detection, semantic segmentation, and action recognition.
	[4]	Detection	Camera-based detection and tracking for UAVs.
	[1]	Recognition	Review of deep learning-based OR algorithms.
	[5]	Recognition	OR Voice assisting visually impaired individuals.
	[6]	Detection	Review of object detection techniques using images/videos with DL methods.
	[7]	Detection	Deep learning methods for detecting objects in images.
	[8]	Recognition	Overview of brain-inspired models for visual OR.
EM Wave-Based	[10,23,25]	Detection and Recognition	Review of (deep learning) techniques applied to radar signals for target detection/recognition.
	[16,17]	Recognition	Signal Processing on UWB radar signals for OR.
	[21,22]	Recognition	Terahertz optical machine learning to recognize hidden objects.
	[27,28,29,30,31]	Recognition	Radar-based OR through high resolution range profile.
	[32]	Recognition	Moving target classification using 1D CNN based on Doppler spectrogram.
	[33]	Recognition	Microwave-based OR in the 2–8 GHz range.
	[34]	Recognition	Automotive Radar for millimeter wave OR.
	[24]	Detection	GPR application to detect buried explosive objects.
	[2]	Characterization	GPR application to obtain some features of buried objects.

In this work, electromagnetic backscattering over 1 GHz band around 38 GHz is collected from several items belonging to different classes, and is stored in a dataset to train and test a 1D CNN aimed at item recognition. Actually, a similar approach has been considered in [33,34], but with some clear differences. First, OR is carried out herein at 38 GHz, i.e., somewhere in the middle between the frequencies considered in [34] (77 GHz) and in [33] (below 6 GHz). Second, the training and test datasets here consist of items’ backscattering responses in the frequency domain, whereas in [34], they are collected in the time domain, and in [33], in the range-angle domain. Interestingly, object recognition based on items’ spectral features is also acknowledged as a recommended investigation technique in [33]. Finally, and most importantly, four and seven types of different objects have been considered in [33] and [34], respectively, but each class included only a single element. This means that they have investigated to what extent electromagnetic features can help in discriminating a specific item $x_{0}$ belonging to a class $C_{X}$ from another specific item $y_{0}$ belonging to a different class $C_{Y}$. Nevertheless, no insight is provided about the recognition skill when each class includes more objects similar to each other but not exactly equal, i.e., in case $C_{X}=\left\{x_{0},x_{1},\ldots ,x_{n}\right\}$ and $C_{Y}=\left\{y_{0},y_{1},\ldots ,y_{m}\right\}$. The task addressed in this work is therefore twofold: (i) understanding that $x_{i}$ and $y_{j}$, ∀i,j, belong to different classes, and (ii) labeling $x_{i}$ and $x_{j}$, ∀i,j, as elements of the same class. In particular, the latter issue represents a new challenge and was not investigated in [33,34]. In general, any classification task typically turns out harder when each class includes multiple items compared to the case when it is limited to a single object.

## 3. Experimental Framework

### 3.1. Measurement Equipment

**Figure 2:**
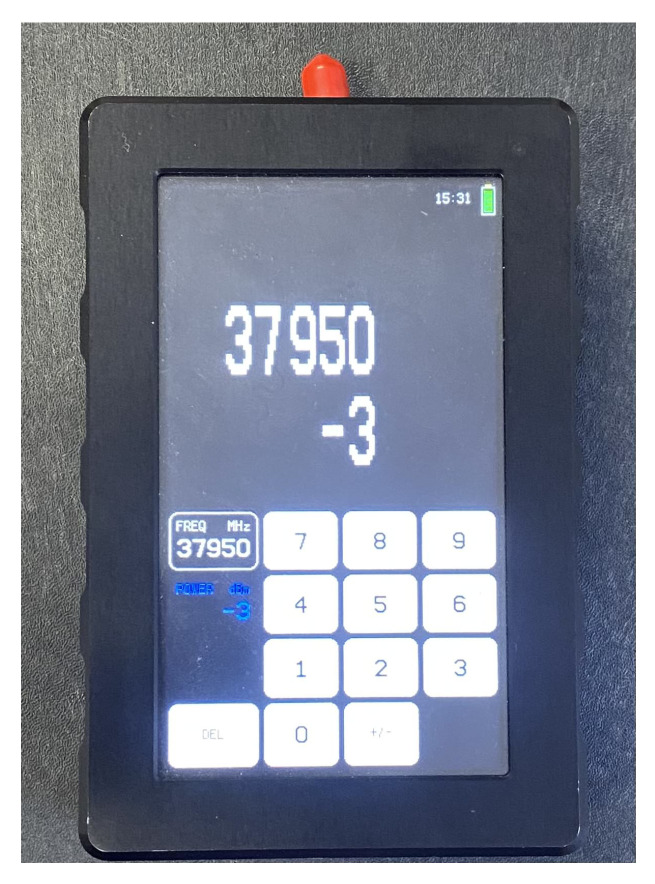
Signal generator.

**Figure 3:**
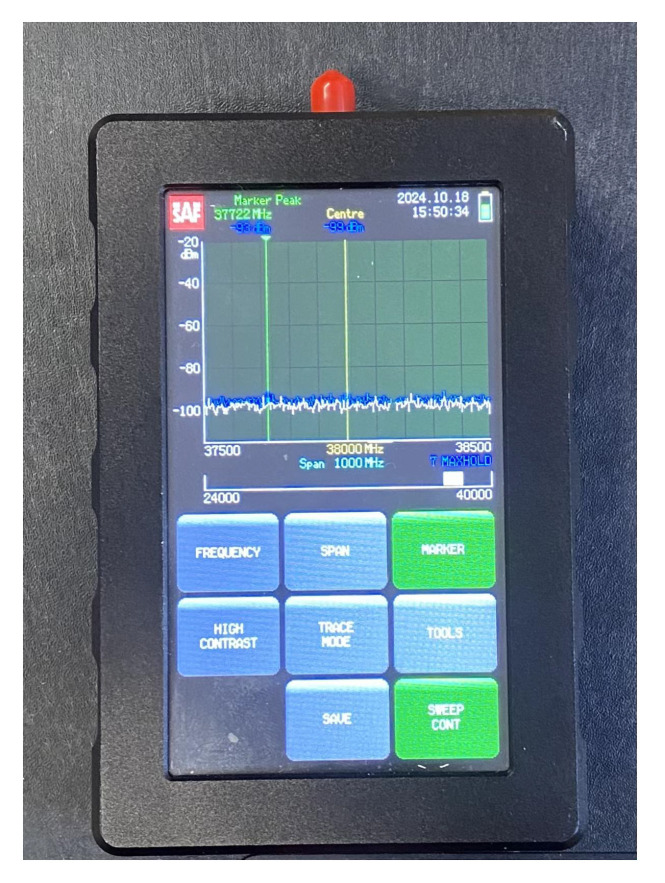
Spectrum analyzer.

**Figure 4:**
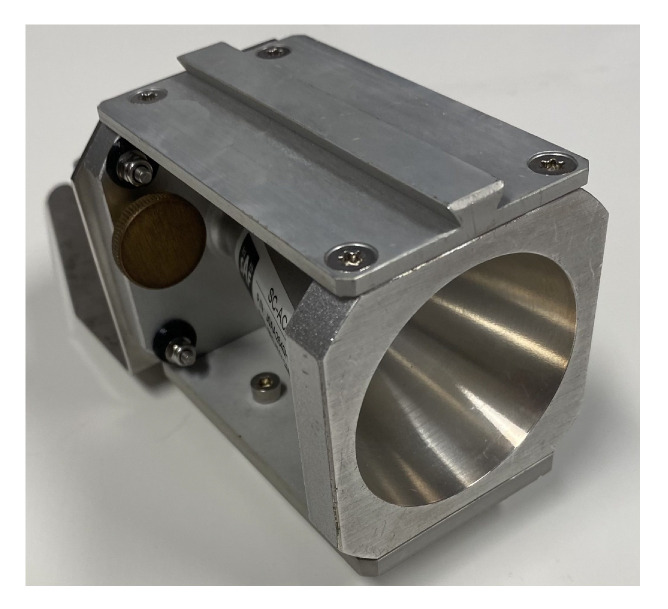
Horn antenna.

The experimental setup consisted of a portable signal generator (SG) and a portable spectrum analyzer (SA), both equipped with conical horn antennas. Some general technical details about the equipment are briefly reported in the following list:

- **Signal Generator**: Wireless signals have been transmitted by the J0SSAG14 portable and light SG from SAF Tehnika [35], which can work in the 26–40 GHz band with an overall radiated power up to 5 dBm (Figure 2).
- **Spectrum Analyzer**: An SAF Tehnika J0SSAP14 compact SA [35] represents the receiving end of the mmWave wireless link. It can also detect signals over the 26–40 GHz band with a sensitivity of about −100 dBm. The sweep time is 0.5 s at a frequency span equal to 100 MHz (Figure 3).
- **Conical Horn Antennas**: These also operate across the frequency band from 26 GHz to 40 GHz, where the power gain ranges from 20.5 dBi to 21.5 dBi. The corresponding half-power beam-width is equal to about 13.5 deg. Vertical polarization was considered during the whole measurement activity (Figure 4).

### 3.2. Object Classes

**Figure 5:**
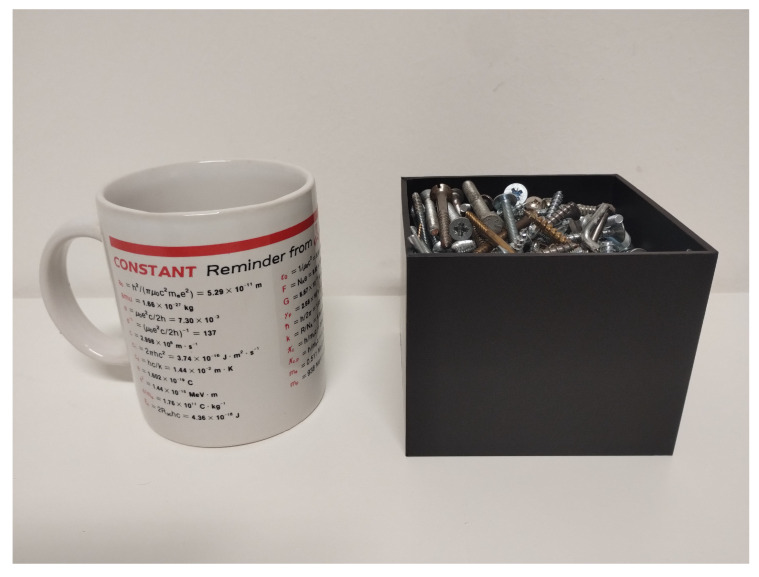
Considered items for OR.

At this stage of the activity, the ML-based approach to OR is applied to two different classes of items:

- **Ceramic Mugs (CMs)**: A total of 18 different mugs, varying in size and shape, were considered (Figure 5). Each mug was measured three times, resulting in 54 measured frequency responses.
- **Box of Screws (SBs)**: A cardboard box was filled with screws and then emptied 18 times (Figure 5), i.e., corresponding to a different deployment of the screws every time, and therefore to a different backscattered signal to some extent. Again, measurements were repeated 3 times for each realization, thus resulting in 54 measured frequency signals overall.

The choice of the items’ classes was mainly driven by a few simple requirements: to ensure that their collection and use could be conveniently managed, objects had to be immediately available in multiple realizations, and also possibly light, cheap, and easy to move. This automatically excluded bulky items such as cars and motorbikes [34], as well as the PlayStation 4 DualShock wireless game controller considered in [33], which was clearly out of the ordinary.

Measurements were also carried out with no object in front of the antennas, and the corresponding collected data are herein referred to as “no object” (NO). Measurements without items were repeated 27 times throughout the time spent to collect data from CMs and SBs. To ensure that spurious signals undesirably received from the environment had limited impact on the measurement repeatability, their intensity had to be lower than the noise threshold of the receiving SA, or at least (much) weaker than the backscattered signals collected from CMs and SBs.

Although electromagnetic backscattering essentially occurs at any frequency, the properties of the target items may affect the choice of the system working frequency. In fact, the size of the item should be conveniently equal to the footprint of the transmitting and receiving antennas on it in order to trigger and collect backscattering from the whole item body, but limiting at the same time the amount of electromagnetic noise coming from the background environment. Therefore, smaller objects call for greater directivity, which, in general, entails larger frequency. Moreover, stronger surface scattering is expected at larger frequency, whereas possible scattering stemming from the inside of the item might benefit from lower frequency. Frequency tuning could therefore also consider the outer and inner structure of the object.

### 3.3. Measurement Procedure and Data Collection

**Figure 6:**
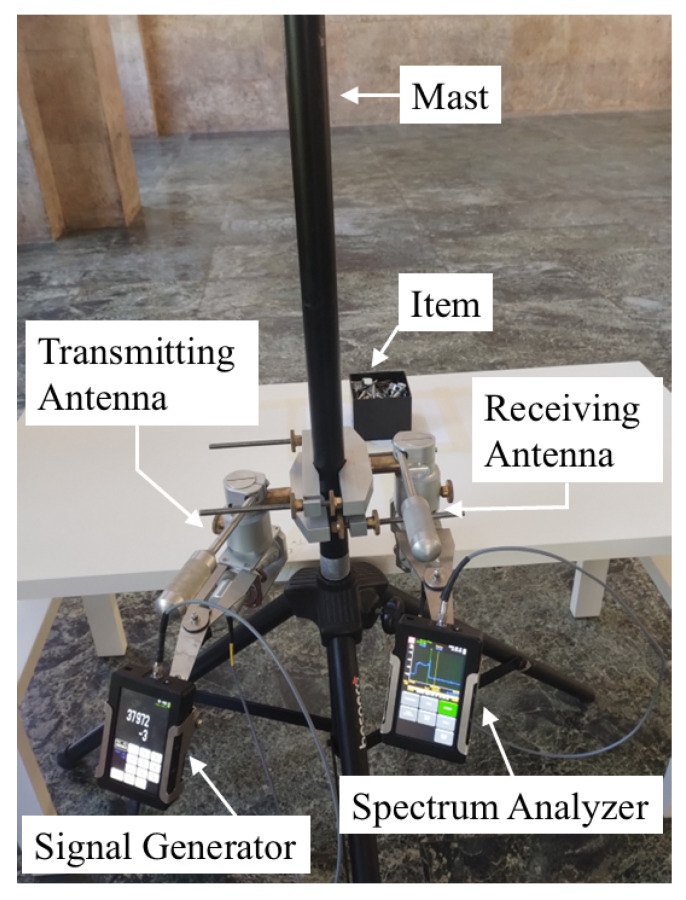
Measurement setup.

**Figure 7:**
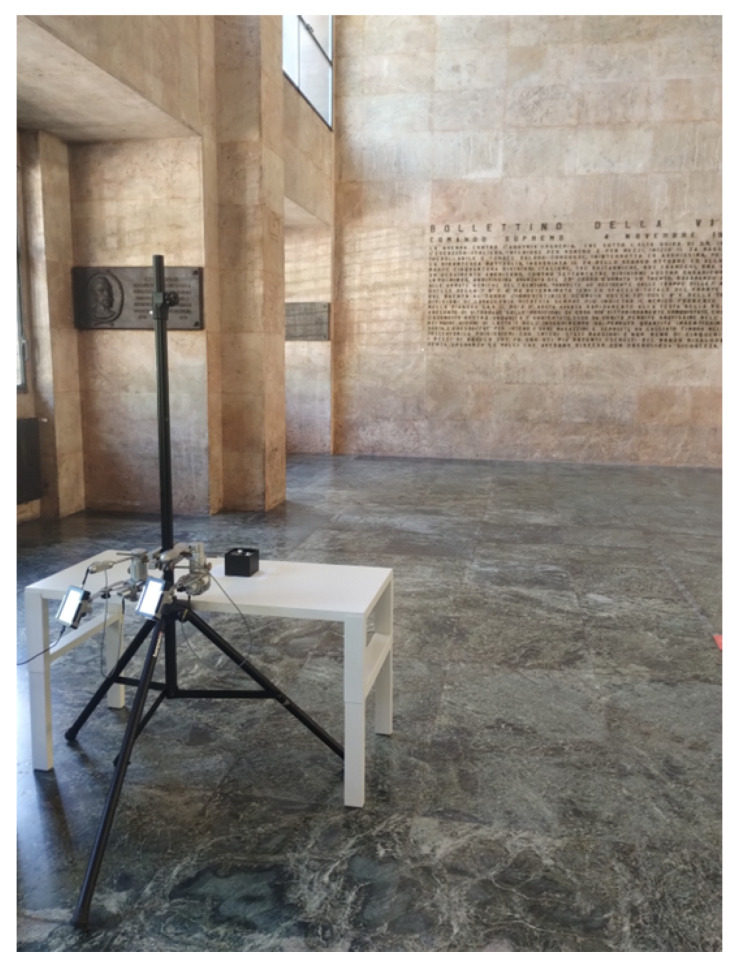
Measurement environment.

The measurement setup is shown in Figure 6, where the transmitting and the receiving antennas, held up on the same mast together with the SG and the SA, are pointed toward the item placed on a wooden table in front of them at a distance of about 40 cm. The signal from the SG feeding the transmitting antenna had a power equal to −3 dBm and a frequency sweeping the 1 GHz band from 37.5 GHz to 38.5 GHz, which sounds fairly consistent with the items’ sizes according to previous considerations, with 40 MHz increments. The radiated signal impinges on the object and triggers a backscattered wave that is then detected by the receiving antenna. The SA finally records the corresponding received signal strength (RSS) for each frequency sample. Since each item affects the properties of the backscattered wave in a different way, the final collected frequency responses represent a set of characteristic imprints that can drive the recognition process. Since the transmitter and the receiver are not synchronized, the time the SG dwells on each frequency was set equal to the sweeping time of the SA over the 1 GHz bandwidth; this means that a new RSS value was achieved at every sweep round. Therefore, the time required to collect the whole frequency response from a single item amounted to 125 s. Of course, synchronization between the transmitting and the receiving stage (e.g., by using a vectorial network analyzer) would greatly reduce the frequency scanning time. In order to limit possible multipath interference from the environment, the measurement activities were carried out in a large, empty hall, where possible sources of additional scattering (besides the target item) were fairly far away from the equipment (Figure 7).

**Figure 8:**
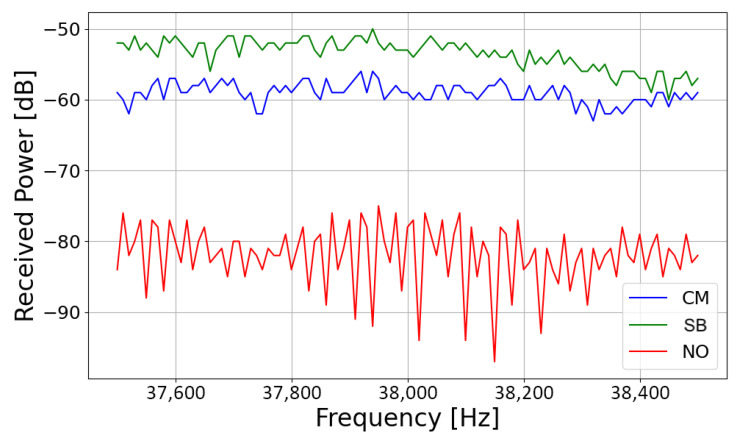
Example of measurement results.

Figure 8 shows an example of received frequency response over the target 1 GHz band for the three considered use cases (CM, SB, and NO). RSS values collected in the presence of an item in front of the antennas (either a CM or an SB) are nearly 20 dB greater than the received signal strength without any objects; that means possible (multipath) interference and/or noise coming from the environment did not affect the measurement to a significant extent. Furthermore, this result also points out that any direct cross-talk between the antennas is either negligible or sufficiently suppressed, further corroborating the physical soundness of the collected measured data.

In order to reduce the sensitivity of the recognition procedure to the distance between the antennas and the item under test, the RSS values collected by the SA have been normalized each time to the maximum value before they have been stored in the final dataset.

## 4. Machine Learning

ML is a branch of artificial intelligence where computers use data to learn patterns and make decisions or predictions without being explicitly programmed. Deep learning (DL), a subfield of ML, has achieved significant breakthroughs in recent years, surpassing human performance in various tasks such as voice-to-text translations, object detection and recognition, anomaly detection, and even recognizing emotions from audio or video recordings [36,37,38,39]. These advancements have opened new frontiers in various fields, including signal processing and wireless communication. While several ML models such as Support Vector Classifiers, XGBoost, and Random Forests can be applied to classification tasks, these methods typically rely on tabular data with handcrafted features. In this work, the input consists of raw electromagnetic signal vectors, where the discriminative patterns are implicitly embedded within the data. For this reason, a 1D CNN is adopted, as it is well suited to automatically extract hierarchical features directly from one-dimensional signals.

### 4.1. Convolutional Neural Networks

Convolutional Neural Networks (CNNs) are a widely adopted deep learning architecture that integrate feature extraction and classification within a single framework [40,41,42]. Unlike traditional ML approaches relying on handcrafted features, CNNs directly learn relevant patterns from raw data, which makes them highly effective in complex recognition tasks [43,44,45]. Although initially developed for image-based applications, CNNs can be adapted to one-dimensional inputs such as time- or frequency-domain signals. However, 2D CNNs are computationally demanding and require large datasets; hence, specialized 1D CNNs have been introduced for efficient processing of sequential data.

### 4.2. One-Dimensional CNNs

**Figure 9:**
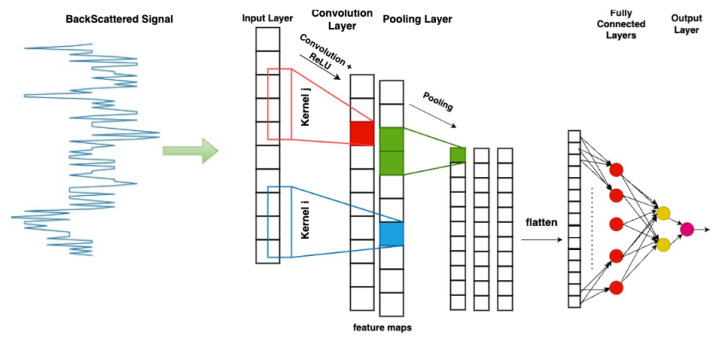
One-dimensional CNN architecture. The red and blue part show a convolution + ReLu action. The green part shows the pooling action. The red and yellow circles in the last part show the fully connected layers.

The 1D CNN framework was first introduced by Kiranyaz et al. in 2015 [18]. One-dimensional CNNs were first conceived to process time-series data, such as electrocardiogram (ECG) signals, directly in their raw form. This capability has enabled significant advancements in signal classification tasks, as 1D CNNs autonomously learn and extract relevant features without requiring extensive preprocessing. Applications span across various domains, including arrhythmia detection from ECG profiles [18,19,20], structural health monitoring [46,47], and fault detection in high-power engines and electrical systems [48,49]. More recent works have also highlighted their effectiveness in identifying damage in mechanical components such as bearings [50,51]. The general architecture of a 1D CNN consists of several different connected layers (Figure 9), shortly outlined below.

**Figure 10:**
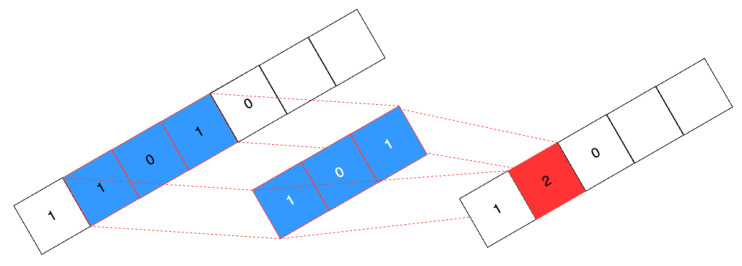
Convolutional layer in 1D CNN.

Convolutional Layers (CLs): They represent in general the core feature of a CNN. These layers apply filters (or kernels) across the input data to capture local patterns. In a 1D CNN, the filters sweep on the input sequence (in this work, on the frequency backscattered responses) and convolution is computed step by step and stored in a final feature map, or activation vector (Figure 9 and Figure 10) [40]. Since convolution is maximized when the input data exactly matches the applied filter, the activation vector keeps track of the occurrence of the filter along the input sequence. This information represents a general, useful feature for classification/recognition, as long as different input signals match the explored kernels. The number of considered filters, as well as the size of each filter, represent hyperparameters of the 1D CNN model. A larger kernel size allows the network to capture broader spectral patterns in the frequency responses, but can also lead to overfitting problems. Increasing the number of kernels enhances the set of different features the model extracts from the backscattered signals, but also corresponds to greater complexity and longer computation time. In the end, 32 kernels having a size equal to 16 turned out to be a fair trade-off between accuracy, complexity, and overfitting risk. After the convolution operation, an activation function (Rectified Linear Unit (ReLU)) is applied to introduce non-linearity into the model [52]. This helps the network capture complex patterns and relationships in the data while addressing the vanishing gradient problem, thereby enhancing the training efficiency and overall model performance [53].

**Figure 11:**
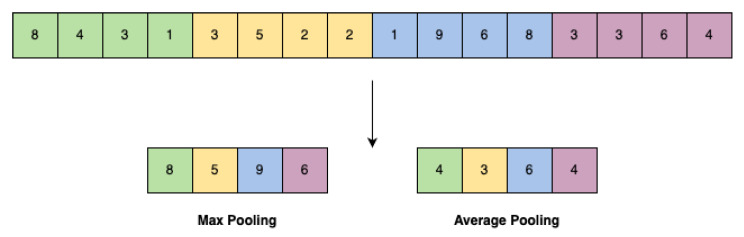
Max pooling and average pooling. Different colors show different part of a singal that maxpooling is applied.

Pooling Layers (PLs): These layers shrink the size of the feature vector (e.g., the sequence of extracted features through convolution) by downsampling it, i.e., reducing the computational complexity and speeding up the classification/recognition process. Max pooling is usually applied, which ultimately aims at removing the lowest entries of the feature vector (Figure 11). This looks like a fair trade-off, as the amount of data is clearly reduced while somehow preserving the most important information about the occurrence rate of the considered kernels throughout the starting input sequence [54]. Nevertheless, average pooling can also be considered (Figure 11). The size of the data blocks where max/average pooling is applied represent another hyperparameter of the convolutional network.

Fully Connected Layers (FCLs): These layers take the high-level feature output from the convolutional and pooling layers and map them to the target outputs, such as class labels, regression values, or other specific tasks [55].

Several other hyperparameters within the model can be tuned to accelerate the learning process and improve accuracy. These include the following [56]:

- **Epochs**: The number of times the entire training dataset is passed through the model. While more epochs can enhance performance, they also increase the risk of overfitting. Even though the same dataset is used in each epoch, the model updates its parameters (weights and biases) after every iteration based on the calculated loss and gradients. Within each epoch, the model refines these parameters, gradually improving its ability to generalize patterns in the data, rather than memorizing it.
- **Learning Rate**: Controls how much the model adjusts its weights during training. A high learning rate speeds up training but may overshoot optimal values, whereas a low rate ensures precise updates but slows convergence.
- **Weight Decay**: A regularization technique that discourages large weight values, helping to prevent overfitting and encouraging simpler models.
- **Batch Size**: The number of training samples used in a single update step. Larger batches provide more stable updates but require more memory, while smaller batches introduce more variability but can generalize better.
- **Early Stopping**: A technique that monitors the iteration process to stop as soon as the step-by-step performance improvement becomes negligible, i.e., when the time required by further iterations is no longer worth the effort.

A grid search strategy was employed to identify effective values for the listed hyperparameters. Different candidate values were considered for each parameter (see Table 2), and all possible combinations were then evaluated. The configuration yielding the best performance on the test set was selected as the final model setup.

To evaluate the performance of the 1D CNN classifier, the confusion matrix is usually taken into account to provide a detailed breakdown of true positives, true negatives, false positives, and false negatives [57]. Based on this, the following key performance indicators (KPIs) are also often considered:

- **Accuracy**: This is simply the probability of correct classification, i.e., the ratio between the number of exactly classified samples and the overall number of samples.
- **Precision**: This measures the proportion of correctly predicted positive observations out of all predictions made as positive. It represents the reliability of the positive classification.
- **Recall**: Also known as sensitivity, it is the probability of positive detection, that is, the probability that a positive sample is correctly classified. It accounts for the model’s ability to detect all relevant instances.
- **F1 Score**: The harmonic mean of precision and recall, the F1 score balances the trade-off between precision and recall, especially when the dataset is imbalanced.
- **Area Under the Curve (AUC)**: This represents the degree of separability between classes, based on the Receiver Operating Characteristic (ROC) curve. It provides insight into how well the model distinguishes between positive and negative classes.

Every considered KPI turns out to be equal to 1 for an ideal, perfect classifier.

### 4.3. One-Dimensional CNN for Object Recognition

The backscattered frequency responses collected with and without items in front of the antennas represent the dataset to set up the 1D CNN aimed at OR. It finally consisted of 135 rows (3 × 18 CM, 3 × 18 SB and 27 NO) and 25 columns (frequency samples from 37.5 to 38.5 GHz with step of 40 MHz). The data were divided into two subsets: 80% for training and 20% for testing. During the training stage, the hyperparameters of the network are as listed in Table 2.

## 5. Results and Discussion

### 5.1. Correlation Analysis

**Figure 12:**
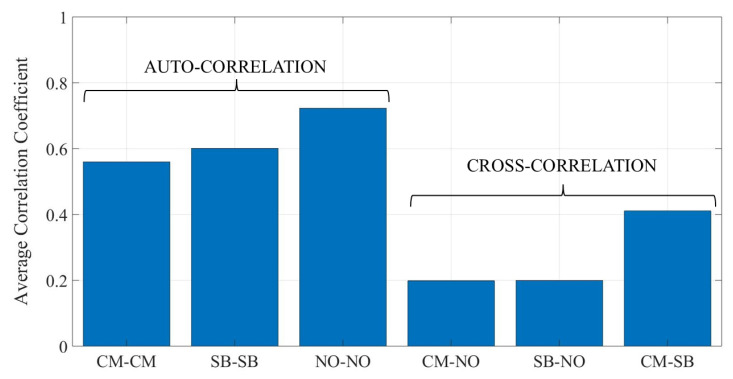
Average auto- and cross-correlation coefficient across the considered classes of items.

Auto- and cross-correlation analyses were first carried out over the raw dataset to investigate the similarity between the signals belonging to the different classes. In particular, the Pearson correlation coefficient $\rho \left(s\left(f\right),v\left(f\right)\right)$ was computed for each pair of frequency responses $s\left(f\right),v\left(f\right)$ stored in the dataset:

(1) $$\rho =\frac{\sum_{i=1}^{N_{f}}\left(s_{i}-E\left[s\right]\right)\cdot \left(v_{i}-E\left[v\right]\right)}{\sqrt{\sum_{i=1}^{N_{f}}{\left(s_{i}-E\left[s\right]\right)}^{2}\cdot {\left(v_{i}-E\left[v\right]\right)}^{2}}},\;with\;s\in X,\;v\in Y\;and\;X,Y\in \left\{CM,SB,NO\right\}$$

where $N_{f}$ is the number of frequency samples collected over the considered bandwidth, $s_{i}=s\left(f_{i}\right)$, $v_{i}=v\left(f_{i}\right)$, *i* = 1,2,…,$N_{f}$, and E[·] is the expected value operator. Then, the mean correlation levels were achieved by averaging the ρ values over each class (auto-correlation, X = Y) and over each pair of classes (cross-correlation, X ≠ Y). The results are reported in Figure 12, and show that auto-correlation is always greater than cross-correlation. This is actually not surprising, as geometrical and electromagnetic properties of items (i.e., the corresponding backscattered imprint) are expected to be quite similar inside the same class, while more different across different classes. In spite of this general trend, auto-/cross-correlation is nevertheless different case by case (Figure 12).

### 5.2. Detection and Recognition Performance

**Figure 13:**
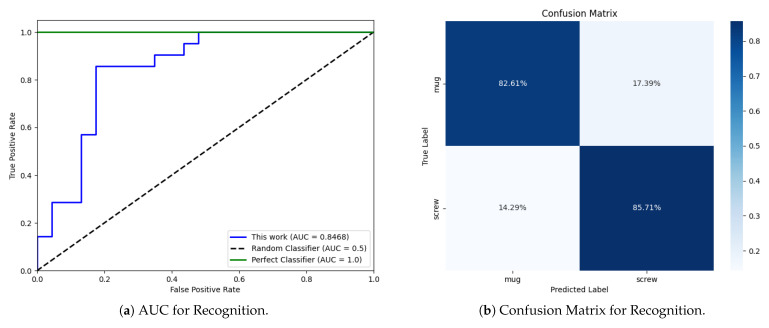
Performance metrics for recognition: (**a**) AUC curve. (**b**) Confusion matrix.

The performance of the 1D CNN model is summed up in Table 3, whereas Figure 13a,b provide further insight related to the recognition task.

The model can perfectly detect the presence of items in front of the antennas, i.e., it behaves as a perfect detector. According to Figure 12, auto-correlation inside the NO class turned out to be far greater than cross-correlation against both the CB and the SB classes. Although a 1D CNN is not just a correlation-based classifier, this clear difference can help explain the perfect object detection skill.

With reference to object recognition, the model performance, while strong, grew slightly worse, with KPIs undergoing some corresponding reduction (Table 3). Overall, the outcome is nonetheless better than blind (or random) recognition (Figure 13a), which would have had KPI values equal to 0.5. This is also supported by Figure 13a, where the AUC curve of the trained 1D CNN model is clearly closer to that of a perfect classifier rather than to the curve of a blind classifier.

The confusion matrix in Figure 13b shows that performance reduction affects both the CM and the SB classes ultimately to the same extent. Every time the 1D CNN is fed with the signal trace backscattered by either a ceramic mug or a box filled with screws, it is correctly labeled with a probability equal to about 82% (Precision). At the same time, the output prediction from the neural network (Recall) turned out to be reliable in approximately 86% of cases regardless of the class. Once again, Figure 12 can contribute to the interpretation of results. Although the auto-correlation values computed over the CM and the SB classes are still greater than their cross-correlation, the difference is not so large, and this can somehow explain the overall performance decrease in recognition compared to detection. Although a 1D CNN is expected to take into account (many) more features than correlation alone—let us stress this aspect again—it is nonetheless interesting to note that the small difference between the auto-correlation of the CM and the SB class in Figure 12 somehow corresponds to the slight performance imbalance in Figure 13b in favour of the SB class.

Although the choice of having more than one item per class has presented a harder challenge compared to [33,34], the final, overall performance is similar, as the test accuracy per class achieved in [33,34] is in the range [87.3% ÷ 92.7%] and [67.5% ÷ 100%], respectively.

### 5.3. Impact of Acquisition Parameters

**Figure 14:**
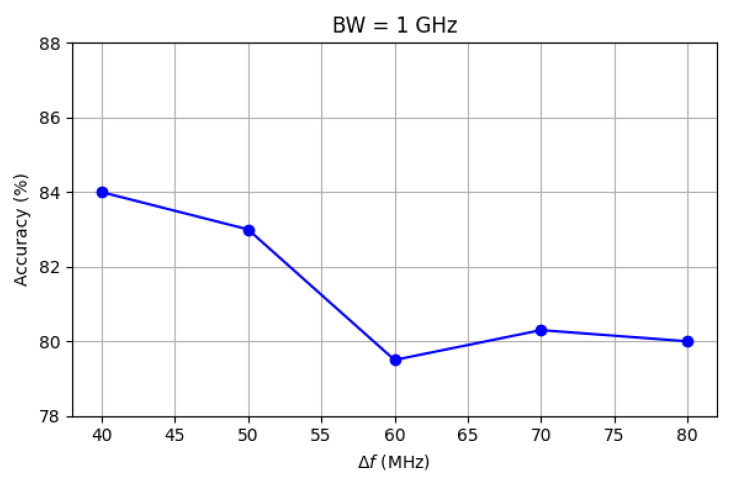
Impact of Δf on test accuracy.

**Figure 15:**
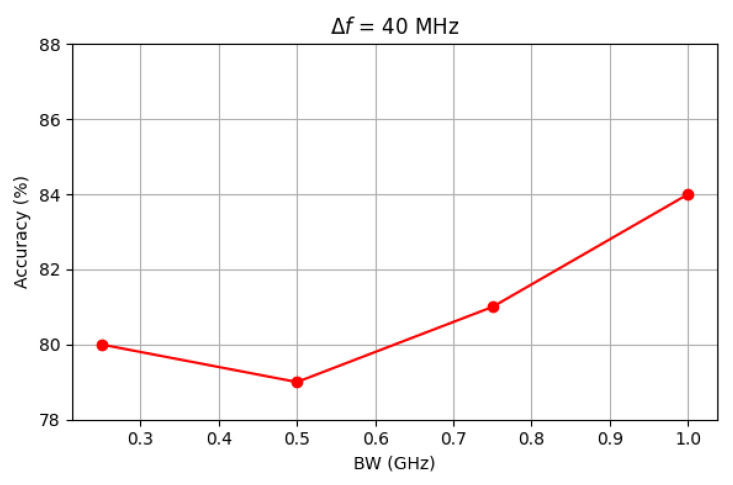
Impact of BW on test accuracy.

With reference to possible, real-time application of OR, a critical factor is the time required to accomplish the recognition task. Since the decision time of the trained 1D CNN is ultimately negligible, any possible latency comes from the frequency scanning for the acquisition of the item backscattered response. In this respect, the importance of synchronization between the transmitting and the receiving stage is clear and has already been stressed. Furthermore, the amplitude of the frequency band under sweep (BW) and the scanning frequency step (Δf) can also affect the final reaction time. Since they can also have an impact on the performance, the sensitivity of the test accuracy to BW and Δf is investigated in Figure 14 and Figure 15.

Increasing the frequency step from 40 MHz to 80 MHz results in a slight decrease in accuracy, which drops from 84% to approximately 80%. A similar reduction is observed when the bandwidth shrinks from 1 GHz to 250 MHz. It can also be noted that in both pictures the accuracy does not steadily decrease across the range considered for Δf and BW. Rather, the trend somehow becomes blurred as Δf and BW approach 40 MHz and 250 MHz, respectively. This is explained by the corresponding reduction in the number of frequency samples ($N_{fs}$) collected for each backscattered signal: if $N_{fs}$ = 25 when Δf = 40 MHz and BW = 1 GHz, it drops to 12 if Δf= 80 MHz and to 6 if BW = 250 MHz. A lower number of collected samples generally corresponds to some lost of information that could have helped in recognition, thus leading to the performance reduction on the average. At the same time, each step of reduction in $N_{fs}$ does not automatically bring a performance reduction, as the level of separability between frequency responses does not necessarily linearly decrease with $N_{fs}$.

### 5.4. A Glance to Open Issues

As already discussed, recognition accuracy assessment with multiple items inside each class represents a new challenge with respect to previous, similar works [33,34]. By contrast, the number of classes was just two (CM, SB). The main reason for this limitation lies in the time required for data collection. The reason was twofold: the lack of synchronization between the transmitter and the receiver, which clearly increased the acquisition time per item, and the restriction to the access to the large university hall where measurements were carried out. It is in fact a public space, with relatively short time periods, and often reserved for exhibitions, a help-desk space for new students or a welcome space for visiting high school students. In conclusion, data collection took more than two weeks overall, which means a large sample size could have hardly been afforded. Nevertheless, increasing the number of classes may represent an interesting improvement.

The choice of the large hall as the measurement site was of course intentional, as a large space with limited environmental noise and interference has clearly helped in the evaluation of the feasibility of the proposed approach. Thus, performance assessment in a real-life scenario (e.g., mimicking an industrial case, where space can be much more restricted) would be a valuable follow-up activity. Finally, accuracy sensitivity to the considered bandwidth and to the frequency sweeping step was investigated across the same frequency (38 GHz), whereas moving to a different frequency band (e.g., 27 GHz) may represent a further, interesting option, as scattering is expected to be significantly frequency-dependent.

## 6. Conclusions

This work presents an effective DL-based framework for object recognition based on electromagnetic wave backscattering, addressing limitations of traditional vision-based systems. By leveraging a 1D Convolutional Neural Network, the model succeeds in the extraction of distinct features from the backscattered signals, achieving perfect accuracy in object detection and satisfactory performance in object recognition, with a classification accuracy equal to 80-85%. With respect to previous, similar investigations, the proposed approach differs in the considered signal frequency, in the domain where data collection is carried out and—most of all—in the size of each class, which is not limited herein to a single item. Additionally, the analysis highlights the trade-offs between accuracy and evaluation speed, depending on the explored bandwidth and the frequency step size. While the execution speed is linearly related to both the bandwidth and the frequency step, their relationship with accuracy is not linear, and this may open pathways to real-time applications, though further investigations are still necessary in this respect. Moreover, future developments may also include a dataset extension to more diverse classes of objects, a performance assessment in real-life scenarios and in different frequency bands, and the use of advanced deep learning architectures to further enhance recognition accuracy.

## Figures and Tables

**Table 2:** One-dimensional CNN hyperparameters.

Description	Value	Note
# Kernels	32	Convolutional Layer
Kernel size	16	
Activation function	ReLU	
Type of pooling	Max	Pooling Layer
Kernel size	2	
# Neurons	50	1st Fully Connected Layer
# Neurons	2	2nd Fully Connected Layer
# Epochs	100	Candidate values: 50, 100, 200
Learning Rate	0.001	Candidate values: 0.0001, 0.001, 0.01, 0.1
Weight Decay	0.001	Candidate values: 0.001, 0.01, 0.1
Batch Size	2	Candidate values: 2, 4, 8
Early Stopping	20	

**Table 3:** Performance of the model for detection and recognition.

Metrics	Accuracy	Precision	Recall	F1 Score	AUC
Detection	100%	100%	100%	100%	100%
Recognition	84%	82%	86%	84%	85%

## Data Availability

The data supporting the findings of this study are available from the corresponding author upon reasonable request.

## Abbreviations

The following abbreviations are used in this manuscript:

ANN	Artificial Neural Network
AUC	Area Under the Curve
BW	Bandwidth
CL	Convolutional Layer
CM	Ceramic Mug
CNN	Convolutional Neural Network
DL	Deep Learning
ECG	Electrocardiogram
FCL	Fully Connected Layer
HRRP	High Resolution Range Profile
KPI	Key Performance Indicator
ML	Machine Learning
NO	No Object
OR	Object Recognition
PL	Pooling Layer
RSS	Received Signal Strength
SA	Spectrum Analyzer
SB	Screw Box
SG	Signal Generator
